# Parkinson’s Disease Wearable Gait Analysis: Kinematic and Dynamic Markers for Diagnosis

**DOI:** 10.3390/s22228773

**Published:** 2022-11-13

**Authors:** Lazzaro di Biase, Luigi Raiano, Maria Letizia Caminiti, Pasquale Maria Pecoraro, Vincenzo Di Lazzaro

**Affiliations:** 1 Research Unit of Neurology, Neurophysiology and Neurobiology, Department of Medicine and Surgery, Università Campus Bio-Medico di Roma, Via Alvaro del Portillo 21, 00128 Roma, Italy; 2 Operative Research Unit of Neurology, Fondazione Policlinico Universitario Campus Bio-Medico, Via Alvaro del Portillo 200, 00128 Rome, Italy; 3 Brain Innovations Lab, Università Campus Bio-Medico di Roma, Via Álvaro del Portillo 21, 00128 Rome, Italy; 4 NeXT: Neurophysiology and Neuroengineering of Human-Technology Interaction Research Unit, Campus Bio-Medico University, 00128 Rome, Italy

**Keywords:** Parkinson’s disease, gait analysis, diagnosis, wearable, kinematic analysis, dynamic analysis

## Abstract

**Introduction:** Gait features differ between Parkinson’s disease (PD) and healthy subjects (HS). Kinematic alterations of gait include reduced gait speed, swing time, and stride length between PD patients and HS. Stride time and swing time variability are increased in PD patients with respect to HS. Additionally, dynamic parameters of asymmetry of gait are significantly different among the two groups. The aim of the present study is to evaluate which kind of gait analysis (dynamic or kinematic) is more informative to discriminate PD and HS gait features. **Methods:** In the present study, we analyzed gait dynamic and kinematic features of 108 PD patients and 88 HS from four cohorts of two datasets. **Results:** Kinematic features showed statistically significant differences among PD patients and HS for gait speed and time Up and Go test and for selected kinematic dispersion indices (standard deviation and interquartile range of swing, stance, and double support time). Dynamic features did not show any statistically significant difference between PD patients and HS. **Discussion:** Despite kinematics features like acceleration being directly proportional to dynamic features like ground reaction force, the results of this study showed the so-called force/rhythm dichotomy since kinematic features were more informative than dynamic ones.

## 1. Introduction

Parkinson’s disease (PD) diagnosis and symptoms monitoring rely mainly on clinical evaluation of the cardinal motor symptoms (bradykinesia, rest tremor, and rigidity) [1,2]. To date, following the in vivo diagnostic criteria [3], the error rate is about 20% [4]. This is mainly due to the lack of objective biomarkers for the in vivo diagnosis of Parkinson’s disease.

**Figure 1:**
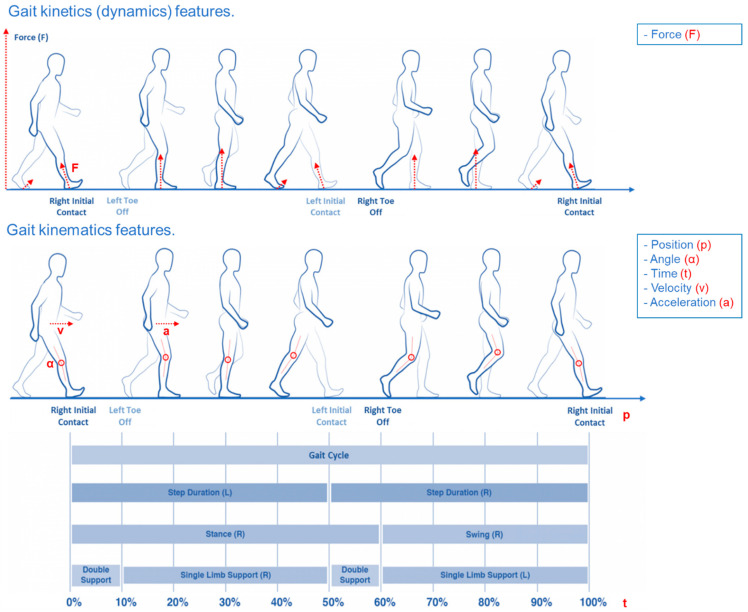
Gait kinetics (**upper** figure) and kinematics features (**lower** figure) (modified under the terms and conditions of the Creative Commons Attribution (CC BY) license from [21]).

Wearable motion sensors are a promising solution to objectively describe PD motor symptoms [5,6], like bradykinesia [7,8,9], rigidity [9,10,11,12], tremor [13,14,15,16], and axial symptoms like gait, balance, and postural issues [17,18,19,20,21]. In addition, the symptom identification process through motion sensors [22] could also improve the therapy management process [23]. Generally speaking, body motion can be analyzed from two different points of view: kinetics (dynamics) analysis, which takes into account the forces that generate the motion and their effect on the body. On the other hand, kinematic analysis, defined as the geometry of motion, describes the movement of the body in terms of position, time, velocity, acceleration, or angle of body segments (Figure 1) [21,24,25].

In literature, kinematic analysis in PD patients showed that the stride variability is increased, and the ability to maintain a steady gait rhythm and a stable, steady walking pattern with minimal stride-to-stride changes is impaired [26,27,28,29]. PD patients show decreased swing time and reduced stride length compared to controls and stride time, i.e., the gait cycle durations is increased with respect to control group but not significantly different, while stride-to-stride variability is increased significantly from the control group [30].

Increased stride variability has been associated with an increased fall risk in older adults in general, as well as in patients with PD [31,32,33], suggesting that this aspect of gait may have clinical utility as an aid in fall risk assessment.

On the dynamics analysis side, the features studied are the forces that cause the motion and their effect on gait. During the stance phase, where feet are in contact with the ground, a level of center of pressure (CoP) is applied the ground reaction force (GRF) which represents the results of gravity force and muscular activation forces counterbalanced by the contact with ground [21,24,25]. Gait dynamics studies have highlighted how some features of GRF vary in different phases of PD, while others are preserved. Components of the GRF are the peak-force at the heel-strike and at toe-off. In the novo early PD there is a delayed heel-strike and an earlier forefoot loading. These parameters seems to be altered independently from the stages of the disease or the pharmacotherapy, instead representing an early marker of the disease [34]. GRF measurement could also be useful to determine gait asymmetry. Su et al. [35] demonstrated how VGRF can reveal the asymmetry of gait by comparing the VGRF of both lower limbs between PD patients and healthy controls. Results showed that PD group has a higher degree of gait asymmetry of the GRF wavelet profile compared to healthy subjects [35]. This metric, compared to conventional asymmetry measures of kinematic features, like step time, stance time, double stance time, or dynamic features like the two peak and the one dep forces of GRF profile, all resulting with higher asymmetry compared to healthy subjects but with lower diagnostic accuracy [35].

The aim of the present study is to evaluate which kind of gait analysis (dynamic or kinematic) can be considered as more informative for discriminating PD and healthy subjects (HS) on the basis of gait features.

## 2. Materials and Methods

### 2.1. Subjects

For the present study, gait data were collected for a total of 108 PD patients and 88 HS from four cohorts [36,37,38] collected in two publicly available datasets [37,38] (Table 1).

For all the four cohorts, inclusion criteria for Parkinson’s disease patients were: idiopathic PD diagnosis, according to the UK Brain Bank criteria [39], and Hoehn and Yahr stage between 2 and 3 [40], a stable antiparkinsonian medication regimen, ability to ambulate independently, and absence of motor fluctuations. Control subjects were included if they did not have Parkinson’s disease or other common exclusion criteria for the Parkinson’s disease group: dementia, clinically significant musculo-skeletal disease, cardio-vascular disease, respiratory disease, other neurological disease, major depression, or uncorrected visual disturbances.

**Figure 2:**
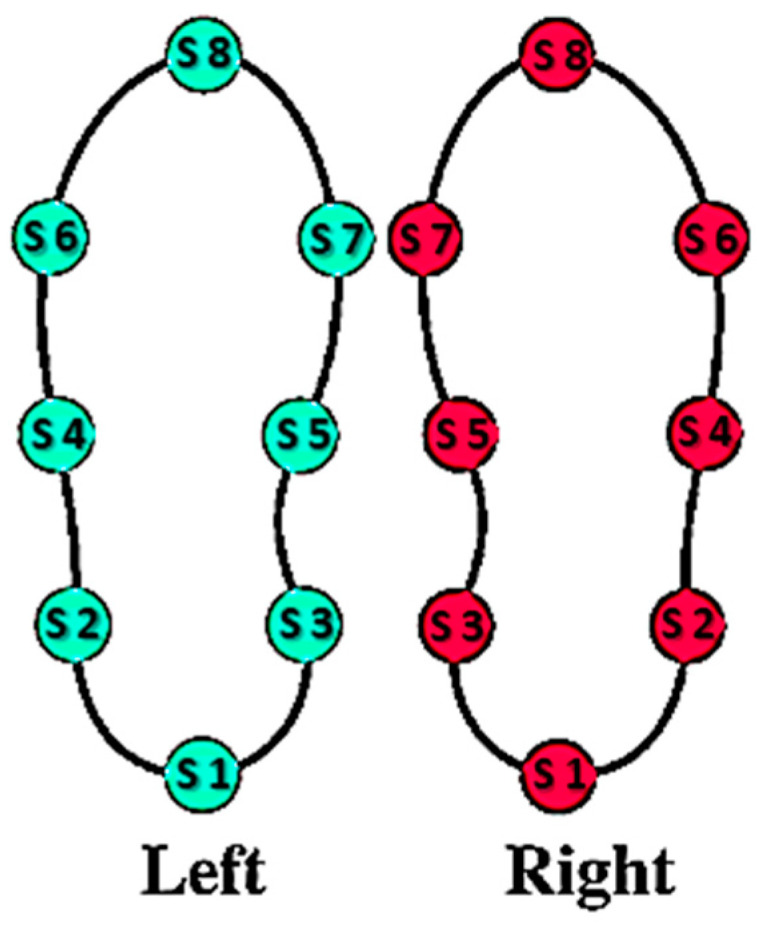
Force-sensitive insole. Each insole placed in subjects’ shoes contains eight pressure-sensitive sensors in order to record the time series of the ground reaction force (GRF), while subjects were asked to walk on level ground.

The first dataset (cohort 1 [36], 2 [37], 3 [30]) was composed of 93 PD patients and 72 HS, while the second dataset (cohort 4) [38] was composed of 15 PD patients and 16 HS. Regarding the demographic analysis cohorts 1, 2, and 3, the 72 HS are age-matched with PD patients. For cohort 4 the 16 HS are younger than PD patients, therefore, although mitigated by the 72 age-matched HS from the other cohorts, we need to take it into account as a possible bias of the study. For all the four cohorts, for both Parkinson’s disease patients and HS, gait-related data were collected through an instrumented force-sensitive insole [43] placed in subjects’ shoes, containing each eight pressure-sensitive sensors (Figure 2), thus allowing the experimenters to record the time series of the GRF while subjects were asked to walk on level ground. In the first and third cohort subjects walked for two minutes, in the second cohort for 100 m (around 80 s), and in the fourth cohort for 5 min. Considering that in each cohort PD and HS walked with the same protocol, and that around 10 m or 10 s of gait recording are sufficient to catch the gait pattern in PD and HS, the data available in the four cohorts are sufficient to describe the gait kinematic and dynamic. However, the inhomogeneity of gait duration protocol across cohorts should be considered as a limit of the present study.

All patients gave informed consent, and the study was approved by local research ethics committees in accordance with the Declaration of Helsinki.

### 2.2. Data Analysis

In the first dataset, only the gait dynamic data were available. Therefore, in order to analyze kinematic data, the recorded GRF signals were used to segment the single gait cycle periods for each patient.

**Figure 3:**
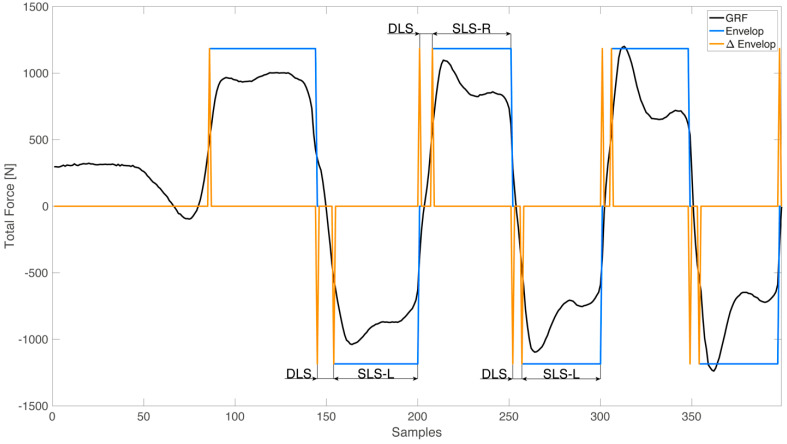
Segmentation of the gait cycle implemented using the differential ground reaction force (GRF) between total right force and total left force. DLS: double limb support, GRF: ground reaction force, SLS-L: left single limb support, SLS-R: right single limb support.

According to Figure 3, the segmentation of the gait cycle was implemented using the differential *Ground Reaction Force* (GRF) (δ) between total right force and total left force:

(1) $$\delta =R_{foot,tot}-L_{foot,tot}\;\;$$

In (Equation (1)), $R_{foot,tot}$ and $L_{foot,tot}$ denote the sum of the forces (expressed in newton) measured by all the sensors embedded in the insole worn under the right foot and the left foot, respectively.

On the basis of δ, each gait cycle for each patient was selected between the first double limb support (DLS) and the left single limb support (SLS-L) (Figure 3).

For each cycle, we computed the following parameters related to the gait:

- Right and Left Stance, expressed both in seconds and as percentage of the stride length;
- Right and Left Swing, expressed both in seconds and as percentage of the stride length;
- Double Limb Supports, expressed both in seconds and as percentage of the stride length
- Right and Left Single Limb Supports, expressed both in seconds and as percentage of the stride length;
- Right and Left Step Duration, expressed both in seconds and as percentage of the stride length;
- Gait velocity expressed in m/s
- Time up and go test expressed in seconds

Such parameters were then averaged along all cycles for each subject.

Moreover, in order to remove single cycle outliers, we compared the duration of each cycle (i.e., stride length) with the average duration ($\bar{SL}$) computed for each subject. To this aim, we marked and then discarded all those cycles whose duration was higher then $\bar{SL}+2\cdot SD\left(SL\right)$, denoting with SD(SL) the standard deviation of the stride length of all cycles.

Considering the second dataset [38,39], two sources of data were available: raw data of the instrumented insoles (containing the whole gait dynamics) and the processed data already containing gait interval parameters (gait kinematics). Therefore, considering the second dataset, no further data manipulation was performed.

For the sake of simplicity, we summarized the main data manipulation steps performed with the two datasets in Table 2.

### 2.3. Dynamic Analysis

In order to estimate the force applied during the gait, we used the raw data of the instrumented insoles from both datasets. However, the data available within the second dataset were not calibrated, i.e., they were expressed in volts. Therefore, in order to compare the data between the two group of subjects, we divided the recorded signals by the maximal output of the electronic system composing the insole, according to [43]. This allowed us to obtain signals expressed in percentage of the maximal detectable force by the insole. For comparing the two groups (PD vs. HS), we computed the following central tendency and dispersion features: (1) mean, (2) standard deviation (SD), (3) median, (4) interquartile range (IQR). We computed such features along the whole trial duration, for each subject and each group, and we used *t*-test analysis to statistically test the difference between the two groups.

### 2.4. Kinematic Analysis

The following gait kinematic parameters were included in the analysis:

- Right and Left Stance, expressed both in seconds and as percentage of the stride length;
- Right and Left Swing, expressed both in seconds and as percentage of the stride length;
- Double Limb Supports, expressed both in seconds and as percentage of the stride length;
- Gait velocity expressed in m/s
- Time up and go test expressed in seconds

Similar to the data analysis presented in Section 2.3 from the raw force data, we computed the following central tendency and dispersion features for the gait kinematics parameters: (1) average (ave), (2) standard deviation (SD), (3) median (med), (4) interquartile range (IQR). We computed such features along the whole trial duration for each subject and each group, and we used t-test analysis to statistically test the difference between the two groups.

For both kinematic and dynamic analysis, Bonferroni correction was applied, considering a correction factor of 50, deriving from the number of dynamic and kinematic parameters. Therefore, the statistically significant value (*p*) threshold is equal to 0.001 (0.05/50).

## 3. Results

### 3.1. Kinematic Analysis

**Figure 4:**
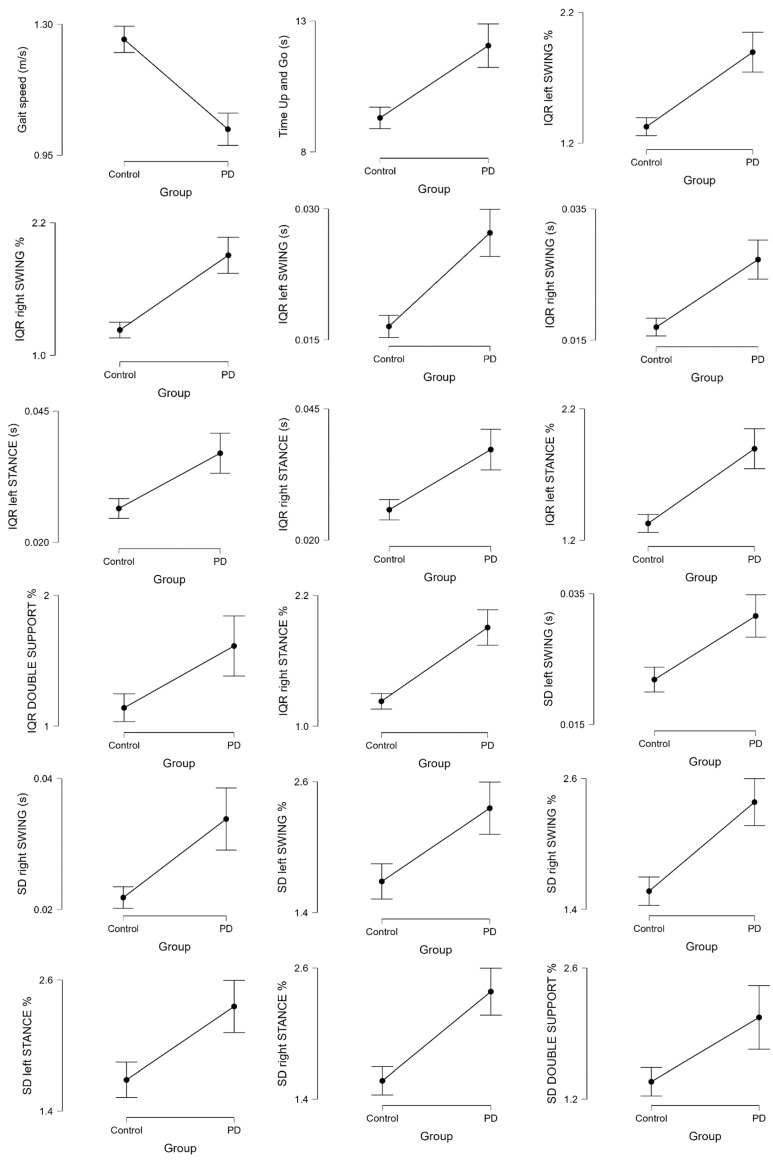
Average values of kinematic central tendency indices (gait speed and Time Up and Go) and dispersion indices of PD patients and HS with significant differences on the *t*-test. IQR = interquartile range. SD = standard deviation.

Considering the central tendency indices related to Gait Speed and Time Up and Go test, *t*-tests showed a significant difference between HS and PD (*p* < 0.001) (Table 3, Figure 4), while all other kinematic central tendency indices *t*-test showed a non-significant difference in HS and PD (Table 2).

Moreover, *t*-tests showed a significant difference in HS and PD (*p* < 0.001) (Table 2, Figure 4) considering the dispersion indices computed for the following parameters:

- Standard deviation (SD) left and right SWING absolute and percentage value
- Standard deviation (SD) left and right STANCE percentage value
- Standard deviation (SD) DOUBLE SUPPORT percentage value
- Interquartile range (IQR) left and right SWING absolute and percentage value
- Interquartile range (IQR) left and right STANCE absolute and percentage value
- Interquartile range (IQR) DOUBLE SUPPORT percentage value

Conversely, for the other kinematic dispersion indices, *t*-test showed a non-significant difference between HS and PD (Table 2).

#### ROC Analysis

**Figure 5:**
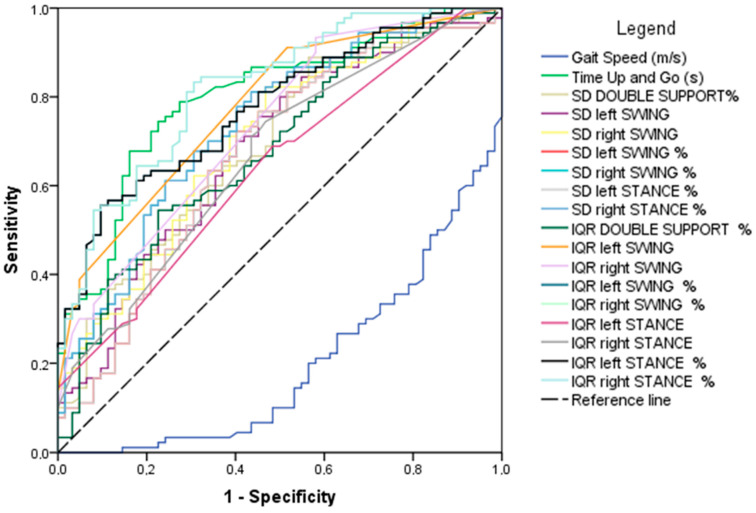
ROC graph of statistically significant kinematic features, considering a diagnosis as a target of PD over HS. IQR: interquartile range, SD: standard deviation.

A ROC analysis was performed for all kinematic values which showed a significant *t*-test difference in HS and PD. It was implemented considering a diagnosis of PD over HS as the target (Figure 5). The value of AUC with upper and lower limits (95% C.I.), the standard error, and the *p* value are listed in Table 4.

As shown in Figure 5 and Table 4, with the exception of “IQR left STANCE”, and “IQR DOUBLE SUPPORT %”, all the kinematic predictors analysed showed a statistically significant ROC AUC value in the discrimination between diagnosis of PD over HS.

### 3.2. Dynamic Analysis

For all the dynamic central and dispersion indices, *t*-test showed a non-significant difference in HS and PD (*p* > 0.001) (Table 5).

**Figure 6:**
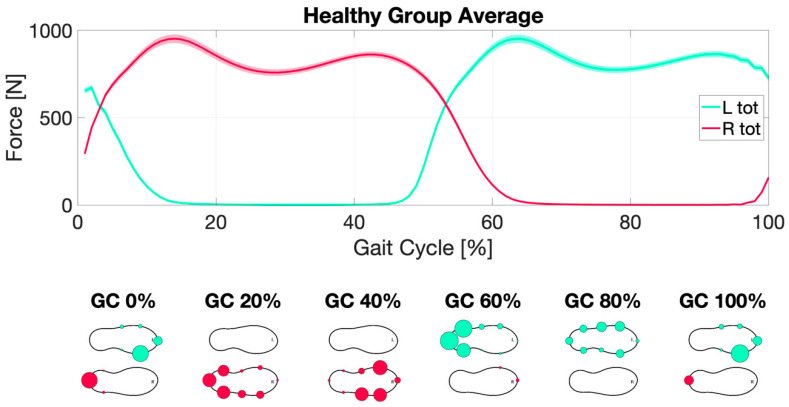
Healthy subjects gait dynamic. Right (R tot) (red line) and Left (L tot) (green line) total force averaged along gait cycles (GC) and subjects over the percentage of completion of the gait cycle (top). The bottom part of the figure represents a graphical visualization of the force measured by the single sensors embedded within the instrumented insole: the larger the circles (right red; left green), the larger the force measured.

**Figure 7:**
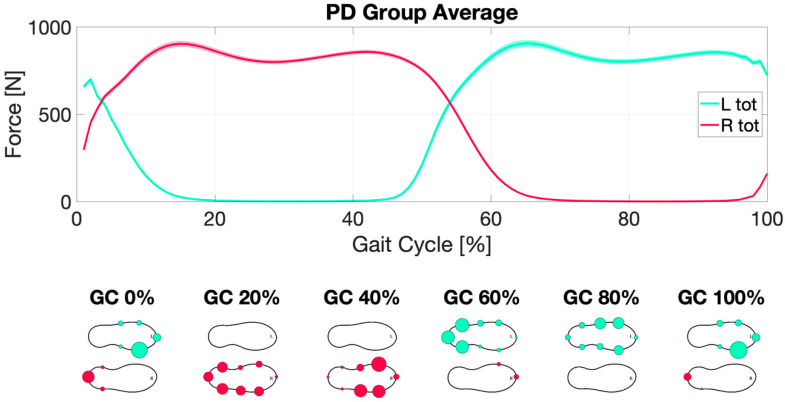
Parkinson’s disease gait dynamic. Right (R tot) (red line) and Left (L tot) (green line) total force averaged along gait cycles (GC) and subjects over the percentage of completion of the gait cycle (top). The bottom part of the figure represents a graphical visualization of the force measured by the single sensors embedded within the instrumented insole: the larger the circles (right red; left green), the larger the force measured.

In Figure 6 and Figure 7 (and Supplementary Video S1), the average gait cycle dynamic, respectively, in HS and PD groups are summarized, showing no difference in gait cycle dynamic profile in the two groups.

## 4. Discussion

Gait features differ between PD patients and HS under normal conditions. In this article we compared the kinematic and dynamic markers of gait between PD patients and HS. The statistical analyses related to the kinematic parameters showed significant differences among PD patients and HS for gait speed and time Up and Go test, and for selected kinematic dispersion indices, with statistically significant ROC AUC values indicating good discrimination ability between the two groups of these parameters. These results are in line with literature data showing an increased stride-to-stride variability in PD patients compared to HS [26,27,28,29,30]. This may reflect mechanisms that underline disease pathology, such as reduced automaticity and damaged locomotor synergies. Indeed, different studies showed that stride variability is reduced by levodopa therapy, demonstrating the role of dopaminergic pathways in the gait rhythmicity [27,28,44,45,46]. Moreover, increased gait variability could be a byproduct of bradykinesia and of a lower gait speed. In literature, no significant increase in stride time variability was observed in healthy elderly subjects, even though they walked significantly slower than young adults [47,48,49]. Several studies aimed to define the relationship between gait speed and stride time variability. Gait speed seems to be related to stride length, stride time, swing time, and stride time variability, with similar relationships in patients with PD and in controls. A U-shaped relationship between stride length variability and gait speed was described when healthy subjects walked on a treadmill [50]. Other studies observed a linear relationship between gait speed and stride time variability, and the range of walking speeds tested and differences in study populations may explain this apparent contradiction [51]. Indeed, mechanical and energy expenditure optimizations may be affected by aging and disease [52]. Interestingly, in a study of young and older adults, it was reported that gait speed did not affect the variability of walking velocity, stride length, or stride time [53]. The increased swing time variability in PD is apparently independent of gait speed. Furthermore, even when patients with PD walk at the same speed as controls, swing time variability is increased in PD [51].

In our study dynamic features did not show any statistically significant difference between PD patients and HS. The reason for the differences between kinematic and dynamic analysis, from which kinematic parameters seem to be more sensitive to identify PD patient features with respect to HS, could be found in the dynamic analysis technique. For dynamic analysis, GRF has several characteristics that make it suitable for gait study. Above all, the acceleration of the center of gravity of the body (COM) is directly proportional to the GRF, which implies that many gait features can be extracted from the GRF. GRF is a continuous signal, unlike kinematic parameters such as oscillation time or stride length, which are considered discrete variables. A great advantage of continuous signals is the possibility of being characterized in terms of time and frequency. However, to simplify the cost and complexity of instrumental devices, only the vertical component of the GRF (VGRF) is usually measured. VGRF is the component of the force with the greatest extent that the ground affects the body, and the majority of dynamic studies are focused on different characteristics of VGRF between patients with PD and controls [35,54]. The result of the present study compared to literature data on dynamic studies showed that to catch a difference between PD and HS a more deep dynamic analysis is necessary, like asymmetry between the two sides [35], or analysis not only of the global GRF but of the dynamics of the individual foot sensors sections (e.g., forefoot heel) [34].

The novelty of the present study is in the direct comparison of the two kinds of gait analysis (dynamic and kinematic). Despite kinematics features like acceleration that are directly proportional to dynamic features like ground reaction force, the results of this study showed the so-called force/rhythm dichotomy, since kinematic features were more informative than dynamic ones. In literature, the two kinds of analysis are very well described, with a lack of a direct comparison between the two on the same data. The limits of the present study, which are related to the source of data that comes from available datasets of previous studies, are the inhomogeneity of gait duration protocol across cohorts and the younger age of HS of cohort 4 with respect to other subjects. Therefore, future clinical trials are needed to confirm these results and additional approaches could be devoted to applying machine learning algorithms to more precisely assess and combine kinematics and dynamics parameters, and weigh the impact of single features.

## Figures and Tables

**Table 1:** Composition of cohorts.

Ref	Cohort	Dataset	Data Source	Group	Subjects Number	Gender	Age (m ± SD)	Hoehn and Yahr (m ± SD)	UPDRS (m ± SD)
[36]	1	1	Movement Disorders Unit at the Tel-Aviv Sourasky Medical Center	PD	29	69% male	71 ± 8	2.3 ± 0.4	33 ± 12
				HS	18	56% male	72 ± 7		
[37]	2	1	Movement Disorders Unit at the Tel Aviv Sourasky Medical Center	PD	29	55% male	67 ± 9	2.4 ± 0.4	25 ± 8
				HS	25	46% male	65 ± 7		
[30]	3	1	Movement Disorders Unit at the Tel-Aviv Sourasky Medical Center.	PD	35	63% male	62 ± 9	2.1 ± 0.2	36 ± 11
				HS	29	62% male	58 ± 7		
[38]	4	2	Neurology Outpatient Clinic at Massachusetts General Hospital.	PD	15	67% male	67 ± 11	2.8 ± 0.9	
				HS	16	13% male	39 ± 19		

Legend: HS: healthy subjects, Hoehn and Yahr scale [41] is a clinical scale that describes the PD stage from 1 (unilateral body involvement) to 5 (confinement to bed or wheelchair), IQR: interquartile range, med: median, PD: Parkinson’s disease patients, m ± SD: mean ± standard deviation, UPDRS total: Unified Parkinson’s Disease Rating Scale [42] is a clinical scale used to follow Parkinson’s disease symptoms during the disease course.

**Table 2:** Overview of the main data manipulation steps.

First Dataset Cohort 1-2-3	Second Dataset Cohort 4
Type of Data Available	
raw data of the instrumented insoles (gait dynamics)	raw data of the instrumented insoles (gait dynamics) gait interval parameters (gait kinematics)
Data Manipulation	
Kinematic Analysis: Calculation of the differential ground reaction force (δ). Extrapolation of the DLS and SLS parameters. Calculation of the gait cycles and the main kinematic parameters (see Figure 1) Normalization with respect to gait cycle.	Kinematic Analysis: Kinematic parameters were already available; thus no further data manipulation was performed.
Dynamic Analysis: Dynamic data already available, thus no further data manipulation was performed.	Dynamic Analysis: Dynamic data available but expressed as raw signals of the instrumented insoles (expressed in volt). Thus, a normalization of the raw signals was performed in order with respect the maximal output of the electronic system composing the insole (according to [43]).

**Table 3:** Kinematic central tendency and dispersion indices.

Variables			Group	N	Average	Standard Deviation	t	df	*p* Value
Kinematic	central tendency indices	Gait Speed (m/s)	HS	88	1.260	0.166	8.278	194	<0.001 *
			PD	108	1.019	0.227			
		Time Up and Go (s)	HS	62	9.300	1.604	−5.187	150	<0.001 *
			PD	90	12.056	3.962			
		Ave left SWING	HS	88	0.442	0.040	0.361	194	0.719
			PD	108	0.439	0.046			
		Ave right SWING	HS	88	0.443	0.041	1.158	194	0.248
			PD	108	0.435	0.047			
		Ave left SWING %	HS	88	41.804	3.143	1.951	194	0.053
			PD	108	40.781	4.018			
		Ave right SWING %	HS	88	41.916	3.488	2.682	194	0.008
			PD	108	40.395	4.284			
		Ave left STANCE	HS	88	0.618	0.071	−2.127	194	0.035
			PD	108	0.646	0.109			
		Ave right STANCE	HS	88	0.616	0.074	−2.431	194	0.016
			PD	108	0.650	0.109			
		Ave left STANCE %	HS	88	58.196	3.143	−1.951	194	0.053
			PD	108	59.219	4.018			
		Ave right STANCE %	HS	88	58.084	3.488	−2.682	194	0.008
			PD	108	59.605	4.284			
		Ave DOUBLE SUPPORT	HS	88	0.115	0.095	−1.106	194	0.270
			PD	108	0.133	0.120			
		Ave DOUBLE SUPPORT %	HS	88	10.681	8.528	−0.808	194	0.420
			PD	108	11.734	9.492			
		Med left SWING	HS	88	0.441	0.040	0.123	194	0.902
			PD	108	0.440	0.048			
		Med right SWING	HS	88	0.442	0.041	0.963	194	0.337
			PD	108	0.436	0.047			
		Med left SWING %	HS	88	41.999	3.195	1.844	194	0.067
			PD	108	41.029	4.003			
		Med right SWING %	HS	88	42.064	3.487	2.502	194	0.013
			PD	108	40.655	4.240			
		Med left STANCE	HS	88	0.611	0.069	−2.043	194	0.042
			PD	108	0.638	0.106			
		Med right STANCE	HS	88	0.611	0.073	−2.303	194	0.022
			PD	108	0.642	0.106			
		Med left STANCE %	HS	88	58.001	3.195	−1.844	194	0.067
			PD	108	58.971	4.003			
		Med right STANCE %	HS	88	57.936	3.487	−2.502	194	0.013
			PD	108	59.345	4.240			
		Med DOUBLE SUPPORT	HS	88	0.113	0.094	−0.943	194	0.347
			PD	108	0.127	0.110			
		Med DOUBLE SUPPORT%	HS	88	10.483	8.518	−0.742	194	0.459
			PD	108	11.441	9.368			
	dispersion indices	SD left SWING	HS	88	0.022	0.009	−4.851	194	<0.001 *
			PD	108	0.032	0.017			
		SD right SWING	HS	88	0.022	0.008	−4.357	194	<0.001 *
			PD	108	0.034	0.025			
		SD left SWING %	HS	88	1.686	0.762	−4.400	194	<0.001 *
			PD	108	2.357	1.254			
		SD right SWING %	HS	88	1.568	0.613	−6.093	194	<0.001 *
			PD	108	2.383	1.127			
		SD left STANCE	HS	88	0.035	0.016	−1.640	194	0.103
			PD	108	0.065	0.170			
		SD right STANCE	HS	88	0.033	0.014	−1.736	194	0.084
			PD	108	0.058	0.135			
		SD left STANCE %	HS	88	1.686	0.762	−4.400	194	<0.001 *
			PD	108	2.357	1.254			
		SD right STANCE %	HS	88	1.568	0.613	−6.093	194	<0.001 *
			PD	108	2.383	1.127			
		SD DOUBLE SUPPORT	HS	88	0.019	0.015	−1.441	194	0.151
			PD	108	0.045	0.171			
		SD DOUBLE SUPPORT %	HS	88	1.386	0.718	−3.396	194	<0.001 *
			PD	108	2.072	1.780			
		IQR left SWING	HS	88	0.017	0.006	−6.651	194	<0.001 *
			PD	108	0.027	0.014			
		IQR right SWING	HS	88	0.017	0.006	−5.821	194	<0.001 *
			PD	108	0.027	0.016			
		IQR left SWING %	HS	88	1.326	0.323	−6.279	194	<0.001 *
			PD	108	1.896	0.799			
		IQR right SWING %	HS	88	1.229	0.337	−7.009	194	<0.001 *
			PD	108	1.905	0.852			
		IQR left STANCE	HS	88	0.026	0.009	−4.577	194	<0.001 *
			PD	108	0.037	0.020			
		IQR right STANCE	HS	88	0.026	0.009	−4.902	194	<0.001 *
			PD	108	0.037	0.020			
		IQR left STANCE %	HS	88	1.326	0.323	−6.279	194	<0.001 *
			PD	108	1.896	0.799			
		IQR right STANCE %	HS	88	1.229	0.337	−7.009	194	<0.001 *
			PD	108	1.905	0.852			
		IQR DOUBLE SUPPORT	HS	88	0.013	0.008	−2.875	194	0.004
			PD	108	0.018	0.014			
		IQR_DOUBLE_SUPPORT %	HS	88	1.141	0.502	−3.446	194	<0.001 *
			PD	108	1.613	1.203			

Legend: ave: average, HS: healthy subjects, IQR: interquartile range, med: median, PD: Parkinson’s disease patients, SD: standard deviation, *: *t*-test statistically significant *p*-value.

**Table 4:** ROC analysis of statistically significant kinematic features.

Variables	AUC	Standard Error	*p* Value	Lower Limit	Upper Limit
Gait Speed (m/s)	0.200	0.035	<0.001 *	0.130	0.269
Time Up and Go (s)	0.801	0.036	<0.001 *	0.730	0.872
SD left SWING	0.682	0.044	<0.001 *	0.595	0.768
SD right SWING	0.703	0.043	<0.001 *	0.620	0.787
SD left SWING %	0.674	0.045	<0.001 *	0.585	0.763
SD right SWING %	0.740	0.041	<0.001 *	0.660	0.819
SD left STANCE %	0.674	0.045	<0.001 *	0.585	0.763
SD right STANCE %	0.740	0.041	<0.001 *	0.660	0.819
SD DOUBLE SUPPORT%	0.643	0.039	<0.001 *	0.566	0.720
IQR left SWING	0.778	0.037	<0.001 *	0.704	0.851
IQR right SWING	0.733	0.041	<0.001 *	0.654	0.813
IQR left SWING %	0.776	0.037	<0.001 *	0.703	0.848
IQR right SWING %	0.820	0.034	<0.001 *	0.754	0.886
IQR left STANCE	0.639	0.045	0.0036	0.551	0.727
IQR right STANCE	0.667	0.044	<0.001 *	0.580	0.754
IQR left STANCE %	0.776	0.037	<0.001 *	0.703	0.848
IQR right STANCE %	0.820	0.034	<0.001 *	0.754	0.886
IQR DOUBLE SUPPORT %	0.634	0.040	0.0012	0.556	0.712

Legend: IQR: interquartile range, SD: standard deviation, * ROC: statistically significant *p*-value.

**Table 5:** Dynamic central tendency and dispersion indices.

Variables			Group	N	Average	Standard Deviation	t	df	*p* Value
dynamic	central tendency indices	Ave Force left	HS	88	372.346	181.982	−0.813	194	0.417
			PD	108	392.385	162.683			
		Ave Force right	HS	88	369.036	181.877	−1.044	194	0.298
			PD	108	394.455	158.804			
		Med Force left	HS	88	467.395	235.065	−0.537	194	0.592
			PD	108	484.518	210.938			
		Med Force right	HS	88	459.291	235.530	−1.025	194	0.307
			PD	108	491.887	209.364			
	dispersion indices	SD Force left	HS	88	324.871	160.322	−0.561	194	0.576
			PD	108	336.977	141.656			
		SD Force right	HS	88	324.106	160.490	−0.608	194	0.544
			PD	108	337.011	136.732			
		IQR Force left	HS	88	671.779	332.736	−0.764	194	0.446
			PD	108	706.230	297.570			
		IQR Force right	HS	88	671.246	332.261	−0.877	194	0.382
			PD	108	710.175	288.892			

Legend: ave: average, HS: healthy subjects, IQR: interquartile range, med: median, PD: Parkinson’s disease patients, SD: standard deviation.

## Data Availability

Not applicable.

## Supplementary Materials

The following supporting information can be downloaded at: https://www.mdpi.com/article/10.3390/s22228773/s1, Video S1: Average gait cycle dynamic, in healthy subjects (HS) and Parkinson's disease (PD) group.
